# Inherited 2q23.1 microdeletions involving the *MBD5* locus

**DOI:** 10.1002/mgg3.316

**Published:** 2017-08-08

**Authors:** Shereen Tadros, Rubin Wang, Jonathan J. Waters, Christine Waterman, Amanda L. Collins, Morag N. Collinson, Joo W. Ahn, Dragana Josifova, Ravi Chetan, Ajith Kumar

**Affiliations:** ^1^ North East Thames Regional Genetics Service Great Ormond Street Hospital London WC1N 3JH UK; ^2^ Wessex Regional Genetics Laboratory Salisbury NHS Foundation Trust Odstock Road Salisbury SP2 8BJ UK; ^3^ Wessex Clinical Genetics Service Princess Anne Hospital Mailpoint 627 Southampton SO16 5YA UK; ^4^ South East Thames Regional Genetics Service Guy's Hospital Great Maze Pond London SE1 9RT UK; ^5^ Department of Paediatrics Southend University Hospital Westcliff on Sea SS0 0RY UK

**Keywords:** 2q23.1, *MBD5*, microdeletions, mosaicism

## Abstract

**Background:**

Microdeletions of 2q23.1 disrupting *MBD5* and loss of function mutations of *MBD5* cause *MBD5‐*Associated Neurodevelopmental disorders (MAND). Nearly all reported patients have been isolated cases of de novo origin.

**Methods:**

This study investigates three families with inherited *MBD5* mutations from three different Regional Genetics Centres in the UK.

**Results:**

Two of the parents in the study had *MBD5* deletions in a mosaic form. The parent with an *MBD5* deletion in an apparently nonmosaic form has a psychiatric disorder in the absence of developmental delay or dysmorphism.

**Conclusions:**

Inherited forms of *MBD5* deletions are rare, but do occur, especially in a mosaic form. The phenotypic spectrum of MAND may be wider than previously thought.

## Introduction

With the use of CGH or SNP microarray, several loci with reciprocal microdeletions and microduplications associated with developmental delay have been described (Vissers et al. 2003). 2q23.1 microdeletion causes a phenotype consisting of intellectual disability, seizures, variable dysmorphic features, and behavioral problems including autistic spectrum disorder (Vissers et al. 2003; de Vries et al. 2005; Jaillard et al. 2009; Van Bon et al. 2010; Motobayashi et al. 2012). The clinical spectrum has been designated *MBD5*‐Associated Neurodevelopmental Disorders (MAND) (Mullegama and Elsea 2016). Haploinsufficiency of *MBD5* (Methyl‐CpG‐binding Domain 5) (OMIM 611472) has been shown to be the cause of the phenotype associated with 2q23.1 microdeletion (Williams et al. 2010; Talkowski et al. 2011).

DNA methylation has an important role in the control of gene activity. A group of proteins selectively recognize and bind to methylated CpGs. These proteins have a conserved domain of approximately 70 residues known as the Methyl‐CpG‐binding domain. The members of this family include the Rett syndrome gene *MECP2* and *MBD5* (Roloff et al. 2003).

In addition to deletions involving the 2q23.1 locus, frameshift mutations in *MBD5* have been identified with an overlapping phenotype (Kleefstra et al. 2012; Carvill et al. 2013). Disruption of *Mbd5* in mice causes a phenotype consistent with 2q23.1 microdeletion syndrome (Camarena et al. 2014).

Nearly all cases of 2q23.1 deletions reported in literature have arisen de novo. An affected sib pair was reported by Van Bon et al. (2010).

## Materials and Methods

### Ethical compliance

This report has met with requirements for consent and ethical compliance.

### Patients: clinical characterization

The index patients were referred to the regional Clinical Genetics department with concerns regarding developmental delay/learning difficulties or seizures. They were assessed by a Clinical Geneticist.

### DNA extraction

DNA extraction was performed using AutoGenFlex STAR 3000**,** FlexiGene DNA AGF3000 Kit (Qiagen, Valencia, CA, USA) (Family 1) and using an automated Chemagic blood DNA kit (PerkinElmer, Waltham, MA, USA) (Families 2 and 3).

### Array analysis

*MBD5* deletions were detected by array analysis using different array platforms in each contributing laboratory. Array was performed using CytoScan^®^ 750K SNP array and then scanned on an Affymetrix GeneChip (GCS3000) Scanner. Chromosome Analysis Suite (Chas) 2.0.0.195 was used for feature extraction (both Affymetrix Inc., Santa Clara, CA, USA). Normalized data were processed using Infoquant Fusion v6.0 software (Infoquant, London, UK) with duplicate threshold log2 ratios of +0.4 (gain) and −0.4 (loss) (Family 1). Oligonucleotide array comparative genomic hybridization (array‐CGH) was performed using the Oxford Gene Technologies (OGT, Oxford, UK) International Standard Cytogenomic Array Consortium (ISCA) custom 8 × 60K array, manufactured by Agilent Technologies (Agilent Technologies, Santa Clara, CA). Promega pooled control DNA was used as a reference. The results were analyzed using OGT CytoSure Interpret Software version 3.4.6 (Family 2). Array‐CGH was performed using the Agilent 8 × 60K custom designed oligoarrays (AMADID 028469) with approximately 44,000 probes across the genome. Random primer labeling and hybridization were carried out with patient versus patient sex‐matched reference DNA. Images were acquired using an Agilent scanner (Agilent Technologies, Santa Clara, CA, USA). Data were processed with Feature Extraction (v9.5.3.1) software (Agilent Technologies), and results were analyzed with the CGH Analytics software (v3.5.14; Agilent Technologies) using the ADM2 algorithm and a three‐point filter (Family 3).

Detected copy number gains or losses were compared to in‐house databases of previously reported CNVs and publicly available CNV databases.

### Confirmation and follow‐up

#### Real‐time quantitative polymerase chain reaction

Primers for qPCR were designed online using publicly available software (http://primer3plus.com/cgi-bin/dev/primer3plus.cgi) and then subjected to BLAT searches on http://genome.ucsc.edu to verify genomic position and sequence homology. Target regions were screened for single‐nucleotide polymorphisms using https://secure.ngrl.org.uk/SNPCheck/snpcheck.htm. After amplification and labeling, qPCR was performed using the StepOnePlus Real‐Time PCR System (Applied Biosystems, Warrington, UK). StepOne Software v2.3 was used for analysis. The differences between test and reference samples were calculated by quantitation comparative Ct (∆∆Ct) (Family 1).

#### Fluorescence in situ hybridization (FISH)

FISH on peripheral blood metaphases using commercial probes BACs RP11‐264M11, RP11‐350D5, RP11‐567F11 (BlueGenome) from the deleted 2q23.1‐region, was performed using standard procedures (Families 2 and 3).

## Results

In Family 1, microarray identified a small deletion within 2q23.1 which includes intron 2 and exon 3 from the gene *MBD5* (OMIM *611472) with a minimum size of 0.158 Mb (arr [hg19] 2q23.1 (148,813,424‐148,971,600)x1) and a maximum size of 0.173 Mb (arr [hg19] 2q23.1 (148,806,704‐148,978,888)x1). Using qPCR, Relative Quantitation (RQ) values for the proband and her mother, respectively, against normal pooled control DNA were approximately 0.5, which indicated a copy number loss in this region. Parental qPCR analysis demonstrated that the 2q23.1 copy number loss was maternal in origin. There was no evidence of mosaicism in the maternal sample as judged by qPCR. However, this does not exclude the possibility of mosaicism identifiable using another technique or in another tissue sample.

In Family 2, array CGH analysis showed a deletion involving genes *ORC4* and *MBD5* from exon1 to intron5 with a minimum size of 0.363 Mb (arr[hg19] 2q23.1(148,743,885‐149,106,509)x1)and a maximum size of 0.459 Mb (arr[hg19] 2q23.1(148,691,917‐149,150,814)x1). All three children were tested by microarray. FISH using BACs RP11‐264M11 and RP11‐350D5 confirmed the deletion in the proband and showed the presence of two cell lines in the mother. Fifty per cent (48/97) of metaphases showed a deletion of this region. The remaining metaphases showed a normal signal pattern in the region defined by these probes in the mother (ish del(2)(q23.1q23.1)(RP11‐350D5‐,RP11‐264M11‐)[48]/2q23.1(RP11‐350D5,RP11‐264M11)x2[49]).

**Figure 1:**
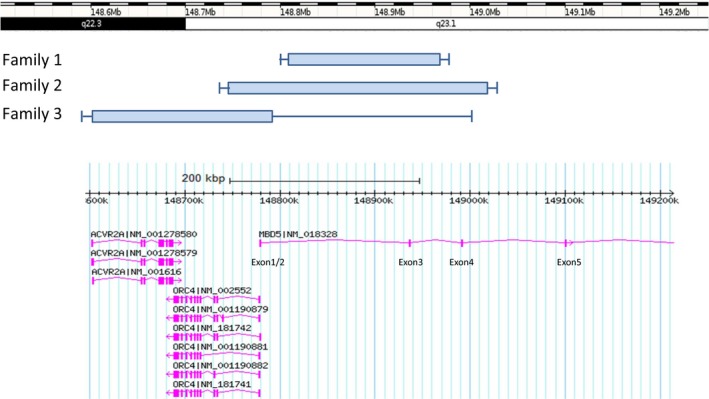
Schematic representation of the minimum (shaded block) and maximum size (line) of copy number losses within 2q22.3 ‐ q23.1 which include MBD5 in Families 1–3 ‐ from Database of Genomic Variants (Build GRCh37: Feb. 2009. Hg19).

In the family 3, array CGH analysis showed a deletion at 2q23.1 including ACVR2A, ORC4, and MBD5 (exon1 to intron2) with a minimum size of 0.188 Mb (arr[hg19] 2q22.3q23.1(148,602,406‐148,790,739)x1)and a maximum size of 0.630 Mb (arr[hg19] 2q23.1(148,372,397‐149,002,433)x1) (Figure 1). Both children were tested by microarray. FISH confirmed the deletion in the proband and demonstrated the deletion in 67% of the metaphase cells examined in the father's sample, ish del(2)(q22.3q23.1)(RP11‐567F11‐)[20]/2q23.1(RP11‐567F11x2)[10] (Table 1, Figure 1).

**Table 1:** Molecular mapping of deletions

Family	Chromosomal segment	Range of proximal and distal breakpoints: maximum and minimum coordinates for each breakpoint shown	Deletion size (Mb)	Genes	MBD5 region identified in copy number loss	Inheritance	ISCN description of mosaicism in parents where identified
1	q23.1	148,806,704‐148,813,424; 148,971,600‐148,978,888	0.158	MBD5	Intron2‐exon3	arr 2q23.1(148,813,424‐148,971,600)x1 mat	N/A
2	q23.1	148,691,917‐148,743,885; 149,106,509‐149,150,814	0.363	ORC4,MBD5	Exton1‐intron4	arr 2q23.1(148,743,885‐149,106,5090x1 mat	ish del(2)(q23.1q23.1)(RP11‐350D5‐,RP11‐264M11‐)[48]/2q23.1(RP11‐350D5,RP11‐264M11)x2[49]).
3	q22.3‐q23.1	148,372,397‐148,602,406; 148,790,739‐149,002,433	0.188	ACVR2A, ORC4,MBD5	Exon1‐intron2	arr 2q22.3q23.1(148,602,406‐148,790,739)x1 pat	ish del(2)(q22.3q23.1)(RP11‐567F11‐)[20]/2q23.1(RP11‐567F11x2)[10]

### Family 1

A 10‐year‐old girl with developmental delay was referred to the clinical genetics department. She had been born prematurely, at 25 weeks and 5 days, and had required ventilation for 3 weeks and a total NICU admission of 3 months. She had associated chronic lung disease, short stature, and myopia. Her developmental problems were attributed to her prematurity.

Further evaluation identified that she had a history of febrile seizures but these had stopped aged seven and she had never had any nonfebrile seizures. Her development had been behind; she sat by 8 months, walked by 18 months and said her first words by 18 months corrected age. She attended a special‐needs school and had difficulties with anxiety and obsessive‐compulsive behaviors. There was no developmental regression.

The clinical examination was unremarkable, but for a height below the 0.4th centile with a weight on the 0.4th centile and head circumference between the 2nd and 9th centiles. She was not dysmorphic.

The maternal history revealed normal development but a background of severe childhood‐onset anxiety and depression with suicidal tendencies and an episode of psychosis requiring psychiatric care. She had completed secondary education and done administrative jobs but found it difficult to hold down jobs because of mental health issues. She was not dysmorphic.

### Family 2

All three children presented with developmental delay and dysmorphic facies.

At age 2, the oldest child was reported to have moderate global developmental delay, particularly in the area of motor skills. He has had seizures from a young age and at 5 years of age was noted to have speech delay. At age 7.5 years, he is attending a special school. He knows his numbers from 1 to 10 and some nursery rhymes. His speech is in basic sentences with some signs. His seizures are coming under control but he is still in nappies and is showing moderate to severe delay overall. He flaps his hand when excited but is not particularly unsteady on his feet. His growth is between the 0.4th and 2nd centiles and he has some dysmorphic features, notably a long philtrum, thin upper lip, and a small lower jaw. He has a multicystic right kidney.

Brain MRI and echocardiogram were both normal.

At age 2, the second child was reported to have moderate global delay and similar features as her brother, with a round face, epicanthic folds, long philtrum, small lower jaw, and prominent forehead. She has never had fits and at age 6.5 years, she is probably the least affected of the three siblings. She is attending a mainstream primary school but is struggling somewhat. However, her reading is said to be improving and her speech is good.

The youngest of the three siblings was also first referred for genetic investigations at the age of 2. She was reported to have developmental delay and dysmorphic facies with epicanthic folds, a long philtrum, and a small lower jaw. She is currently aged 4 years and may be similar to, or slightly more delayed than, her sister. Her speech is slow and basic and parents are hoping that she will attend a mainstream school.

The mother who had the microdeletion in a mosaic form did not have any learning difficulties, dysmorphism, or seizures. She attended secondary school; she is now a carer for her three children.

### Family 3

A 12‐year‐old boy with moderate learning difficulties and autistic spectrum disorder was referred to the clinical genetics department. He was the first child of nonconsanguineous parents, who were both healthy and of normal intelligence. He was born after an uneventful pregnancy, with a birth weight of 4.26 kg. He sat unaided at the age of 1 year, and walked at 18 months of age. At the age of 2 years, he presented with a series of afebrile seizures and was started on medication. This was successfully discontinued at age of 6 years. He has suffered with constipation from a young age, which continues to be medically managed. Otherwise, he was in good general health with normal hearing. He wore glasses to correct myopia. He had mild plagiocephaly, puffiness around his eyes, and epicanthic folds. His height was on the 75th centile with a weight on the 50th centile and head circumference between the 2nd and 9th centiles.

His 18‐month‐old brother had a similar developmental pattern. He sat independently at 11 months, and was unable to pull up to a standing position at the time of clinical review. He was vocal, but not verbal. He, too, was on medication for constipation, although it was less severe than in his older brother. His head circumference lay between the 9th and 25th centile and he had mild brachycephaly. He did not have dysmorphic features.

The father who has mosaicism for the deletion has no history of developmental delay or seizures. He went to an academically selective secondary school and completed high school education. He joined his father's building business and later set up as a window cleaner.

## Discussion

2q23.1 microdeletion syndrome is one of several new reciprocal microdeletion and duplication syndromes that have been delineated since the advent of microarray technology. While these deletions do not have common breakpoints, they do show a common region of overlap, which includes all or part of the gene, *MBD5* implicated as a causal locus for the 2q23.1 microdeletion syndrome (Williams et al. 2010). Patients with intragenic *MBD5* rearrangements and frameshift mutations of *MBD5* are phenotypically indistinguishable from those with 2q23.1 deletions (Kleefstra et al. 2012; Carvill et al. 2013; Camarena et al. 2014; Bonnet et al. 2013). The deletion is highly penetrant and is associated with a broad phenotypic spectrum (*MBD*‐Associated Neurodevelopmental Disorders) (Mullegama and Elsea 2016; Camarena et al. 2014).

Previous reports have found intellectual disability to be a cardinal feature, with speech and language difficulties, seizures, gait abnormalities, stereotypies, and autistic features as prominent in the phenotype. This led to patients being described as pseudo‐Angelman or Rett‐like (Jaillard et al. 2009; Van Bon et al. 2010).

Recently, the phenotype was expanded, with significant behavioral psychopathology reported by Hodge et al. (2013) characterized by anxiety, obsessive‐compulsive disorder, and bipolar disorder.

Although dysmorphic features have been described in patients with microdeletions and mutations of 2q23.1, these are very variable and generally do not allow for a gestalt diagnosis (Jaillard et al. 2009; Van Bon et al. 2010; Williams et al. 2010).

Mullegama et al. described an inherited intronic deletion of MBD5, which does not negatively impact upon mRNA expression and is therefore unlikely to be pathogenic (Mullegama and Elsea 2014). There are no previously reported cases of an affected parent, although a single affected sib‐pair is described by Van Bon et al. (2010) implying dominant inheritance or parental gonadal mosaicism. Further, all previously published cases have included a degree of developmental delay or intellectual disability, which is generally described as moderate to severe. This was not present in the carrier parents described here.

These cases demonstrate instances of inherited 2q23.1 microdeletions involving exons where the parent with the deletion shows no intellectual disability and at least one instance of significant psychiatric morbidity in the absence of learning difficulties or dysmorphism. Mosaicism cannot be excluded in the mother in this family and psychiatric disease is common in the general population. However, given that the daughter who has the deletion in non‐mosaic form has similar mental health issues, this adds to the emerging body of evidence that there exists a psychopathological component to this microdeletion. While the phenotype of *MBD5* haploinsufficiency seems to be fully penetrant, the phenotypic spectrum may be even wider than previously thought.

This series documents two instances of parental mosaicism for a 2q23.1 microdeletion involving *MBD5*. This is significant for sibling recurrence risk of individuals who have an apparent de novo 2q31.1 microdeletion.

## Conflict of Interest

The authors declare no conflict of interest.
